# Chronic obstructive sleep apnea accelerates pulmonary remodeling via TGF-β/miR-185/CoLA1 signaling in a canine model

**DOI:** 10.18632/oncotarget.11296

**Published:** 2016-08-15

**Authors:** Xue Ding, Chengyuan Yu, Yang Liu, Sen Yan, Wenpeng Li, Dingyu Wang, Li Sun, Yu Han, Minghui Li, Song Zhang, Fengxiang Yun, Hongwei Zhao, Yue Li

**Affiliations:** ^1^ Department of Cardiology, the First Affiliated Hospital, Harbin Medical University, Harbin 150001, Heilongjiang Province, P. R. China; ^2^ Key Laboratory of Cardiac Diseases and Heart Failure, Harbin Medical University, Harbin, 150001, Heilongjiang Province, P. R. China; ^3^ Institute of Metabolic Disease, Heilongjiang Academy of Medical Science, Harbin, 150001, Heilongjiang Province, P. R. China

**Keywords:** canine model, chronic obstructive sleep apnea, molecular mechanisms, morphological change, transforming growth factor-beta1, Pathology Section

## Abstract

Chronic obstructive sleep apnea syndrome (OSAS) is considered to be associated with pulmonary diseases. However, the roles and mechanisms of OSA in pulmonary remodeling remain ambiguous. Thus, this study was aimed to elucidate the morphological and mechanical action of OSA in lung remodeling. In the present study, we employed a novel OSA model to mimic the OSA patient and investigate the role of OSA in pulmonary remodeling. We showed that pulmonary artery pressure of OSA group has no significant increased compared with the sham group. Nevertheless, we found that fibrotic tissue was predominantly located around the bronchi and vascular in the lung. Additionally, inflammatory cell infiltration was also detected in the peribonchial and perivascular space. The morphological change in OSA canines was ascertained by ultrastructure variation characterized by mitochondrial swelling, lamellar bodies degeneration and vascular smooth muscle incrassation. Moreover, sympathetic nerve sprouting was markedly increased in OSA group. Mechanistically, we showed that several pivotal proteins including collagen type I(CoLA1), GAP-43, TH and NGF were highly expressed in OSA groups. Furthermore, we found OSA could activated the expression of TGF-β, which subsequently suppressed miR-185 and promoted CoL A1 expression. This signaling cascade leads to pulmonary remodeling. In conclusion, Our data demonstrates that OSA can accelerate the progression of pulmonary remodeling through TGF-β/miR-185/CoLA1 signaling, which would potentially provide therapeutic strategies for chronic OSAS.

## INTRODUCTION

Obstructive sleep apnea syndrome (OSAS) is a disordered breathing with repetitive periods of upper airway collapse during sleep [1]. Mounting evidence has shown that OSA is associated with increased cardiovascular morbidity and mortality [2]. Besides the deteriorative cardiovascular comorbidity, numerous clinical studies also reveal the substantial role of OSA in pulmonary diseases. However, the relationship between OSA and pulmonary dysfunction is still in dispute. For example, previous study indicated that OSA can cause moderate pulmonary hypertension in patients without prerequisite lung disease [3, 4], while some claimed that OSA did not always induce pulmonary hypertension [5]. Additionally, several studies showed OSA frequently associated with chronic obstructive pulmonary disease(COPD). Patients who suffered both these two diseases had a poorer prognosis compared with individual disease [6], which implied a potential tight relationship between OSA and COPD. Nevertheless, in another independent population study, the author found that there was no relationship between OSA and COPD [7].

Based on these controversies, quite a few animal models were adopted to investigate the clinical and mechanistic significance of OSA, including pigs, rats and dogs [8–10]. Nonetheless, these models focused on a short apnea duration, which doesn't conform to the long-term medical history of OSA patients. Recently, we have carried out a novel chronic OSA canine model, which is proved to successfully mimic the OSA patients [11]. Similar with the previous models, it mainly concentrated on the cardiovascular complication, while OSA related pulmonary diseases were less studied.

In this study, we sought to determine the effect of OSA on lung remodeling in a novel canine model as well as the underlying mechanisms.

## RESULTS

### The effect of OSA on pulmonary hypertension in canine

**Figure 1:**
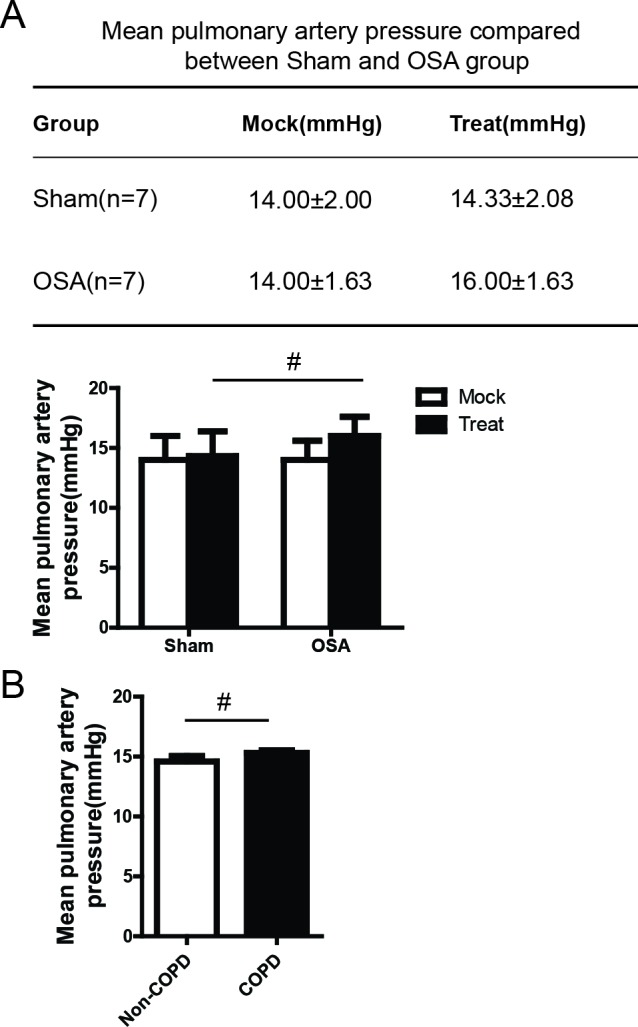
Measurement of mean pulmonary artery pressure between OSA and Sham group before and after anesthesia, n=7 (A) Measurement of mean pulmonary artery pressure in COPD and Non-COPD patients, n=10 (**B**).

To explore the role of OSA in pulmonary remodeling, we first investigated the pulmonary artery pressure by using a novel canine model that we have described previously [11]. Consistent with the previous data, we found that both negative intra-thoracic pressure and negative intra-airway pressure were significantly increased after apnea compared with normal ventilation (data not shown). Meanwhile, lower pH, pO2 and SaO2 whereas higher pCO2 and HCO3- were detected after apnea(data not shown), which indicated that the OSA model was successfully established. We further analyzed the pulmonary artery pressure. Surprisingly we did not find any significant difference of pulmonary artery pressure before and after apnea in OSA group compared with Sham group (Figure 1A). To further confirm this result, we measure the pulmonary artery pressure in 10 COPD patients as well as 10 non-COPD patients. As shown in Figure 1B, we didn't see a significant difference between this two different groups of patients, which is consistent with our data in canine model. Taken together, these findings suggest that OSA doesn't certainly cause pulmonary hypertension.

### Pulmonary fibrosis associated with bronchial remodeling in OSA canine

**Figure 2:**
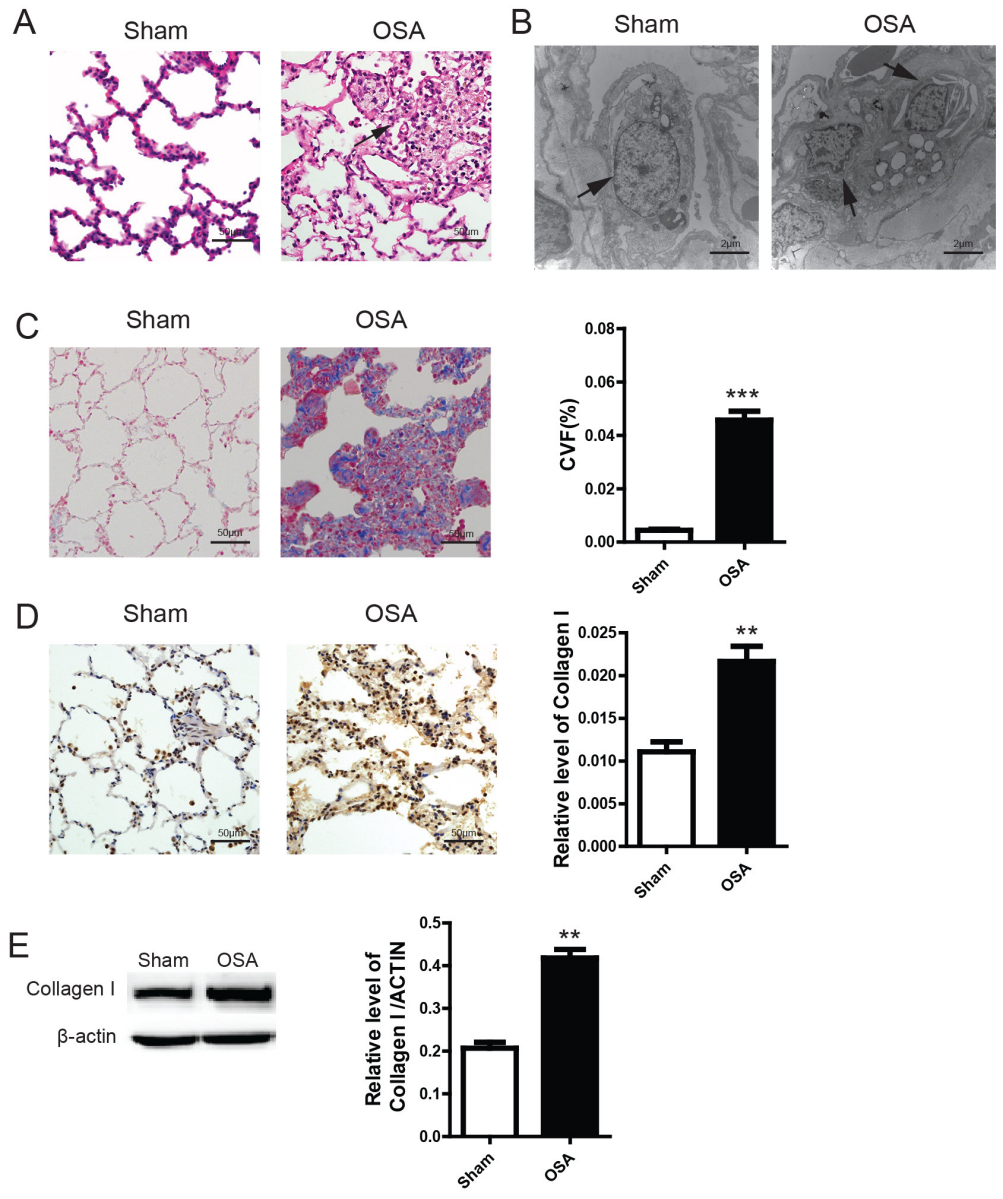
Peribronchial morphological changes after chronic OSA in the lung section of canines (**A**) Hematoxylin and eosin (H&E) staining, scale bar=50μm; (**B**) Transmission electron microscope images of representative alveolar epithelial cell aside the bronchia, the arrow indicates pinocytotic vesicles, vesicular nuclei and nuclear membrane change in the alveolar epithelial cells, scale bar=2μm; (**C**) Masson's trichrome staining of representative peribronchial lung section from different groups and quantitative calculation of collagen volume fraction percentage, scale bar=50μm; (**D**) Immunohistochemistry staining of Collagen type I and Western Blot analysis of protein lysates from the tissue of canines in different groups (**E**), scale bar=50μm.

Next, we sought to determine the morphological alteration of bronchia in OSA canine. As shown in Figure 2A, the alveoli were uniformly arranged with a normal structure in the Sham group. However, the alveoli were disorganized and partially collapsed, which resulted in further alveolar fusion. Meanwhile, a large amount of inflammatory cell infiltration were detected surrounding the bronchia. Further transmission electron microscopy examination (Figure 2B) indicated that the intracellular structure of alveolar epithelial cells was clear and integrate in Sham group. In contrast, pinocytotic vesicles, vesicular nuclei and deranged nuclear membrane were shown in the alveolar epithelial cells of OSA canine. Masson staining showed that fibrotic tissue was extensively located around the bronchia in OSA canine while Sham dogs appeared substantially normal (Figure 2C). Since Collagen type I is a critical protein in fibrosis formation, we next evaluated the expression of Collagen type I using IHC and WB. As shown in Figure 2D and 2E, Collagen type I was significantly increased in OSA group. Thus, our data indicates that OSA could augment pulmonary fibrosis associated with bronchial remodeling.

### Pulmonary vascular remodeling in OSA canine

**Figure 3:**
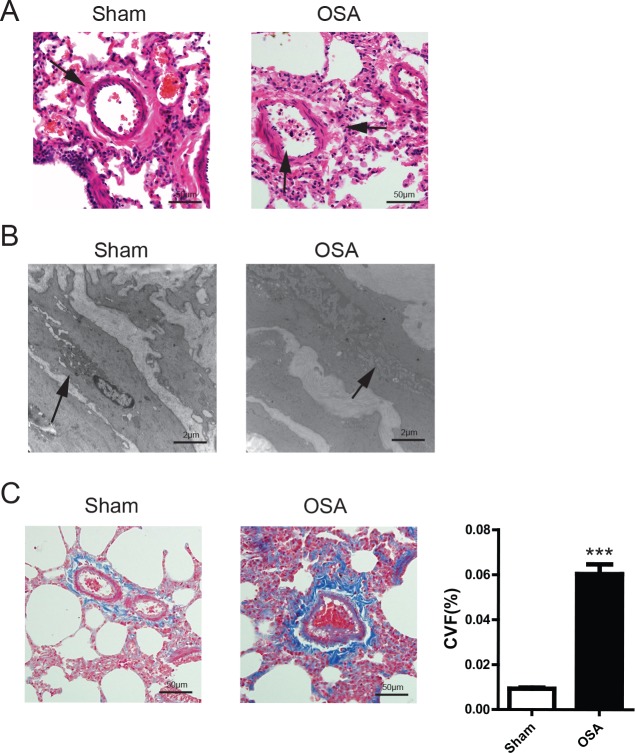
vascular morphological alterations in Sham and OSA canines (**A**) Hematoxylin and eosin (H&E) staining, the arrow indicates the structure change of vascular muscle, scale bar=50μm; (**B**) Transmission electron microscope images of pulmonary artery smooth muscle, the arrow indicates the ultra-structure change of vascular muscle,scale bar=2μm; (**C**) Masson's trichrome staining of representative perivascular lung sections and quantification of collagen volume fraction percentage, scale bar=50μm.

Tough we didn't detect a significant increase of pulmonary artery pressure in OSA canine and given that pulmonary vascular remodeling is the pivotal step of inducing pulmonary hypertension, we were curious to see whether the artery structure was altered after apnea in our canine model. As shown in Figure 3A, the pulmonary artery smooth muscle became distorted with enhanced inflammatory cell infiltration. The morphological change was ascertained by transmission electron microscopy examination characterized by swollen mitochondria and tortuous myofibers alongside the pulmonary artery (Figure 3B). Additionally, Masson staining showed substantive fibrotic tissue dominantly situated in the perivascular space in OSA canine (Figure 3C). Taken together, our results imply that OSA does exacerbate pulmonary vascular remodeling, though without causing pulmonary hypertension.

### Pulmonary autonomic nerve remodeling in OSA canine

**Figure 4:**
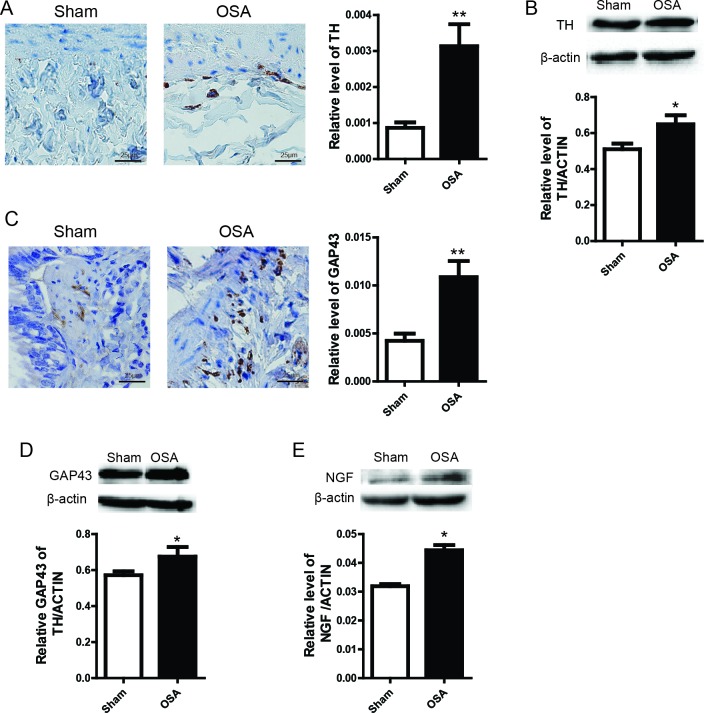
Sympathetic nerve sprouting and protein expression of relevant bio-markers in the lung of Sham and OSA canines (**A**) Immunohistochemistry analysis of sympathetic activity indicator TH and the protein expression (**B**); (**C**) Immunohistochemistry staining of GAP43 and statistical calculation; (**D**, **E**) Protein expression of GAP43 and NGF from the lung tissue collected from the canines. Scale bar=50μm.

It has been well documented that autonomic nerve alteration act as an important part in the pathogenesis of OSA related complication [16]. Thus, we attempted to assess the expression of several already known bio-markers in our OSA model. Consistent with previous reports, we found that tyrosine hydroxylase (TH) was markedly accumulated in OSA group (Figure 4A and 4B). In addition, another important marker, GAP43 and NGF were also highly expressed in OSA group compared with Sham group (Figure 4C, 4D and 4E). Collectively, these data reveal an important role of OSA in autonomic nerve remodeling.

### TGF-β/miR-185/CoLA1 signaling mainly promotes lung remodeling in OSA canine

**Figure 5:**
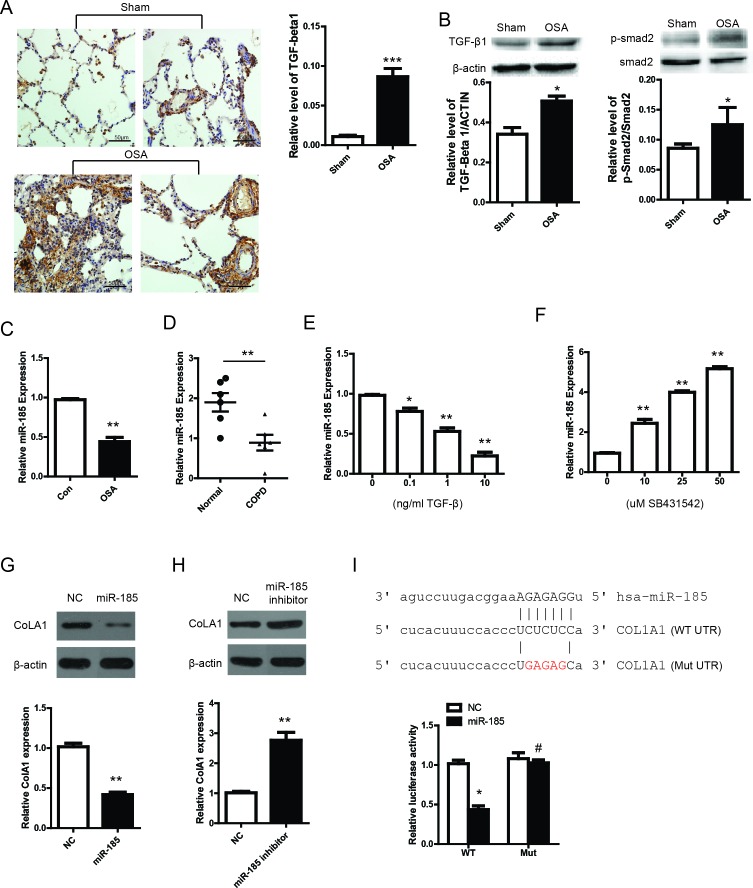
miR-185 is repressed by TGF-β and regulates pulmonary remodeling through CoLA1 (**A**) Immunohistochemistry staining of TGF-β in peribronchial and perivascular lung tissues; (**B**) Protein expression and the statistical quantification of TGF-β/p-Smad2; (**C**) miR-185 expression from lung tissue in Sham and OSA canine; (**D**) miR-185 expression in normal and COPD patient lung tissue from autopsy; miR-185 expression after dose increase of TGF-β (**E**) and TGF-β inhibitor SB431542 (**F**). WB and RT-PCR detection of CoLA1 expression after miR-185 mimics (**G**) or miR-185 inhibitor transfection (**H**). (**I**) Dual luciferase reporter assay of WT and Mut CoLA1 3′ UTR.

To explore the underlying molecular mechanism in OSA induced pulmonary remodeling, we set out to analyze essential proteins of relevant fibrosis pathways. By using IHC staining, we showed that TGF-β and p-Smad2 were notably increased in OSA group (Figure 5A) compared with Sham group, which is also evidenced by WB (Figure 5B). It has been well documented that microRNAs play an important role in lung fibrosis and a group of anti-fibrotic or pro-fibrotic microRNAs were also identified[17]. Interestingly, these experiments were all performed in mice or rat, Thus, we speculated that microRNA might also serve as an important mediator in pulmonary remodeling in the canine model. Based on the literature, we focused on miR-185, which has been reported to be a tumor suppressor [18] and downregulated in idiopathic pulmonary fibrosis[19]. However, it remains unknown whether miR-185 has a role in pulmonary remodeling, especially in a canine model. We firstly detected the expression of miR-185 in Sham and OSA group. As shown in Figure 5C, miR-185 was notably decreased in OSA group, which is accordance with its expression in COPD patient (Figure 5D). Next, we sought to determine why miR-185 was repressed in OSA canine. It has been reported that TGF-β enhanced lung fibrosis through inhibiting or augmenting several microRNAs expression[20]. Then, we suspected whether TGF-β could affect the expression of miR-185. As shown in Figure 5E and 5F, miR-185 was decreased by dose dependent TGF-β treatment and increased by dose dependent TGF-β inhibition. Through TargetScan and miRanda, we tried to search the possible target gene of miR-185. Surprisingly, we found CoLA1 is one of the targets. To validate whether CoLA1 is targeted by miR-185. We transfected primary pulmonary fibroblast cell MRC-5 with miR-185 mimics or inhibitor. As shown in Figure 5G and 5H, CoLA1 was downregulated by miR-185 mimics and upregulated by miR-185 inhibitor. Furthermore, a dual luciferase reporter assay was carried out to examine whether miR-185 directly inhibits CoLA1. We constructed two luciferase reporters by inserting WT or mutated (Mut) 3′ UTR region of CoLA1 gene containing the potential miR-185 binding site. It was found that miR-185 significantly decreased the luciferase activity of the WT reported but not Mut reporter. These results demonstrated that CoLA1 is a direct target of miR-185.

**Figure 6:**
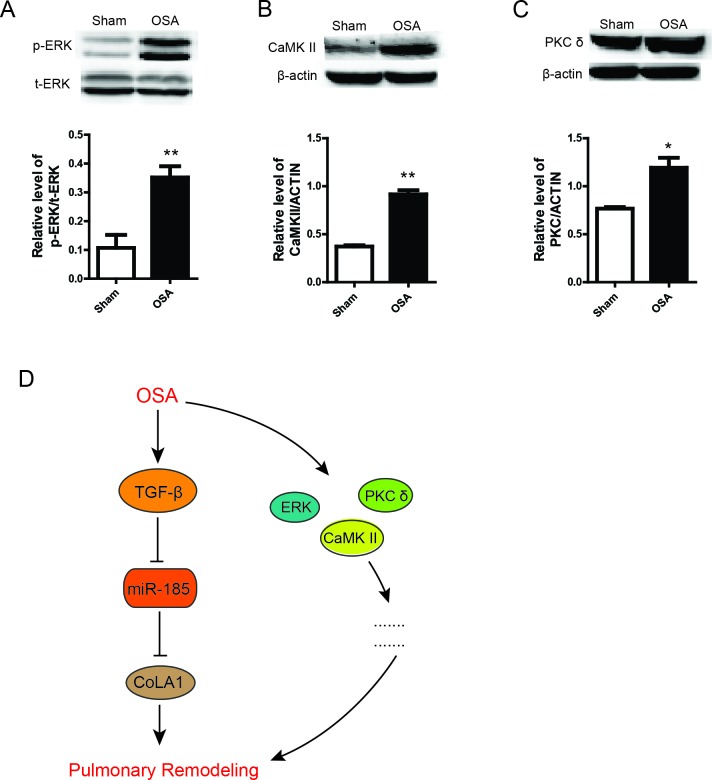
Other possible pathways involved in pulmonary remodeling (**A**-**C**) Protein expression and the statistical quantification of p-ERK, CaMK II and PKC δ respectively. Scale bar=50μm. (**D**) Schematic diagram of the mechanism of this study.

Moreover, we also detected several kinases were remarkably elevated in OSA group, including ERK, CaMK II and PKC δ (Figure 6A, 6B and 6C), which strongly revealed that other signaling could also partly get involved in pulmonary remodeling. In summary, our data uncover a potential role of TGF-β/miR-185/CoLA1signaling mediated and other possible pathways involved modulation of pulmonary remodeling in OSA canine model (Figure 6D).

## DISCUSSION

For decades OSA has been related with extensive cardiovascular comorbidity and even cerebrovascular disease [21], while relatively less attention has been paid to the intrinsic change of lung itself. As a result, the underlying mechanism of OSA induced pulmonary remodeling is less understood. In the present study, by using a novel canine model, we uncovered several primary findings: (i) OSA aggravated pulmonary fibrosis as well as bronchial and vascular remodeling without causing obvious pulmonary hypertension, (ii) OSA enhanced local sympathetic sprouting and changed the expression and distribution of nerve fibers via promoting the expression of TH, GAP43 and NGF, (iii)besides the hyperactivation TGF-β/Smad2 pathway, ERK, CaMK II and PKC δ mediated pathways were also possibly participating in the OSA induced pulmonary remodeling.

In our canine system, apnea stimulation doesn't cause pulmonary hypertension. This finding was unexpected, though it was in accord with some of the previous reports that the prevalence estimate of pulmonary hypertension patient ranged from 17% to 53% [22], which indicates that OSA doesn't definitely lead to pulmonary hypertension. This observation was also consistent with our clinical practice. Intriguingly, we did found a distinct change of pulmonary artery structures in OSA group, which is believed to be highly correlated with pulmonary hypertension. A possible explanation might be that the intervention of apnea in OSA canine is just 12 weeks. The period might not be extended enough to induce pulmonary hypertension, considering the long-term medical history of OSA patients. However, the potential mechanism needs further study.

It has been well documented that inflammation pathway is a crucial mediator in pulmonary remodeling besides oxidative stress response [23–25]. Notably, TGF-β has been reported to stimulate pulmonary fibrosis and inflammation through Bax/Bid/MMP-12 signaling axis [26]. Additionally, several proinflammatory mediators including IL-1α, IL-1β, IL-13 and TNF-α are known to perform a critical role in pulmonary remodeling [27]. In our study, we showed that an extensive recruitment of inflammatory cells predominantly located in peribronchial and perivascular space in OSA canine. This result is consistent with the clinical data that inflammatory cytokines were vastly expressed in the blood of OSA patients. Meanwhile, a strong expression of TGF-β in the lung of OSA canine powerfully enforced the role of TGF-β signaling cascade in pulmonary remodeling. However, further investigation need to be carried out in order to classify the exact type of inflammatory cells.

It is worth noticing that sympathetic nerve activity is markedly increased in pulmonary artery hypertension [16]. Furthermore, TH immunoreactivity is an positive indicator of sympathetic activity according to previous report [28]. Also, GAP43 is an important bio-maker of sympathetic nerve sprouting [29]. Similar with this, NGF deletion could attenuate the sympathetic activity [30]. We therefore hypothesize that there is a tight correlation between sympathetic nerve modulation and OSA induced pulmonary alteration. Based on our data, we did see an exacerbation of sympathetic nerve activity as shown in the IHC staining of TH in OSA canine. Coherently, an increment of GAP43 and NGF expression ascertain the fact that sympathetic nerve remodeling is a reciprocal process with pulmonary remodeling. In one hand, it is the consequence of pulmonary remodeling. In the other hand, it can impair the novel pulmonary function, which further intensifies pulmonary remodeling.

Besides the TGF-β/Smad2 signaling axis, multiple pathways have been reported to role in pulmonary remodeling. For example, Li et al showed that inhibition of MAPK-ERK signaling can reverse high pulmonary blood flow induced pulmonary remodeling [31]. Moreover, CaMK II was found to promote pulmonary remodeling by exerting its enzyme activity on catalyzing a series of serine or threonine residues in other proteins [32]. Meanwhile, PKC δ was proved to be activated in the regulation of lung smooth muscle remodeling [33]. According to our result, to some extent, we demonstrated that these kinases were overexpressed in the lung of OSA canine. The data implies that several possible signaling pathway, at least partially get involved in the regulation of OSA induced pulmonary remodeling.

To sum up, for the first time, our study established a novel canine model to investigate the role of OSA in pulmonary remodeling. Additionally, we elucidated the possible underlying mechanism involved in OSA induced pulmonary remodeling. We believe that all these data could not only open up a pivotal step in discovering the pathogenesis of OSA, but also impart effective therapeutic strategy to the clinical application of OSA patients.

## MATERIALS AND METHODS

### Animals

We have got the approval of the ethic committees of Harbin Medical University to guarantee the progress of all experiments were consistent with the Guide for the Care and Use of Laboratory Animals published by the US National Institutes of Health (8th edition,2011). Fourteen male mongrel dogs weighting between 20~25kg were provided by Experimental Animal Center of the First Affiliated Hospital of Harbin Medical University and raised under a healthy environment according to the laboratory standard. We chose ketamine (5.3 mg/kg, iv), diazepam(0.25 mg/kg, iv) and xylazine (1 mg/kg, iv) as the anaesthetics. We considered the disappearance of the cornel reflex and jaw tone as the success of anesthesia. Dogs were divided into two groups randomly and named sham, OSA group. The dogs in sham group were administrated only anesthesia and tracheal intubation and the others were carried out the establishment of chronic OSA model for 12 weeks.

### OSA stimulation

When the reflex and jaw tone of dogs disappeared, it meant that the anesthesia was successful, next the trachea cannula was done. We clamped the tube in order to induce apnea at the end of exhalation. The protocol of chronic OSA model was administrated from our early research [11]. In short words, we set apnea hypopnea index (AHI) at 6, it meant that the calmping was 1min and resting was 9 mins for the first week. The longer time of blockage, the shorter time of ventilation. AHI was increasing until 10 finally and the blocking time lasted for 5 min. The clamping and loosening procedure was exchange for 4h every other day.

### Measurement of pulmonary artery pressure

Based on the previous study [12], we measured the pulmonary artery pressure successfully according to the protocol. In briefly, we inserted 6F sheath guiding us into the femoral vein percutaneously, and then we can find the pulmonary artery. The sheath, tubing system and pressure transducer were linked together to detect signal and record data as following: pulmonary artery systolic pressure(PASP), pulmonary artery diastolic pressure (PADP) and pulmonary artery mean pressure (PAMP).

### Pathological examination

Lungs were fixed in 4% paraformaldehyde at 4°C for one day and then dehydrated, finally embedded in paraffin. The lung tissues were cut into 5 μm sections and prepared to stain with hematoxyline and eosin (H&E) in order to observe the changes of lung structures using a microscope (Leica DM40000B, Germany). The fibrosis was evaluated by Masson trichrome staining to analysis the changes of collagen using software (Image-pro plus 6.0, Meida Cybernetics LP). The quantitative analysis were called Collagen Volume Fraction (CVF) calculated according to the formula collagen aera/total area×100%.

### Immunohistochemistry

Lung paraffin sections were slided into 3μm-thick, dewaxed in xylol 5min for twice, and then rehydrated in 100% ethanol 5min for twice, 75% ethanol 5min and tap water for 5min. The sections were washed by phosphate-buffered saline (PBS) 3min for twice. 3% Hydrogen peroxide and methanol mixed together and the slides were incubated in order to inactive endogenous peroxidases. Then the slides were put into 0.1% pepsin for antigen retrieval. Next procedure was blocked in protein block solution and incubated with primary antibodies: TGF-β1 (Abcam, USA, 1:400), anti-GAP43 (Abcam, USA, 1:1000), anti-TH (Abcam, USA, 1:300), Collagen I (Bioss, China, 1:300), anti-CollagenIII (Bioss, China, 1:300) at 37°C for 2h, then overnight at 4°C. The slides were washed by PBS, then followed by incubation with biotinylated rabbit anti-IgG (ZSGB-BIO, China) and stained with DAB kit (ZSGB-BIO, China) and hematoxylin. The software (Image-pro plus 6.0, Meida Cybernetics LP) was applied to the analysis of the lung section. We measured the density of positive cells in total lung section.

### Transmission Electron Microscopy (TEM)

Fresh lung tissue (~1mm3) obtained from the canines in sham and OSA group were fixed in 2.5% glutaraldehyde at 4°C overnight. The samples were treated according to previously described [13], The ultramicrotomies were stained and the images were photographed with an electron microscope (JEM-1200, JEOL Ltd, Tokyo, Japan)

### Western blotting assay and reagents

Total protein was isolated from lung tissues using RIPA lysis buffer, 1mM phenylmethylsulfonyl fluoride (PMSF) with protein inhibitor cocktail (Roche Aplied Science, Branford, CT). Protein concentrations were measured by BCA protein assay kit with bovine serum albumin (BSA) as standard (Beyotime, China). The details were followed as previously described [14, 15]. The samples were applied in each well of 10% sodium dodecyl sulfate polyacrylamide gel electrophoresis (SDS-PAGE) to separate proteins. After that, protein was transferred to Polyviny-lidene fluoride (PVDF) membrane using a transfer system and electric field of 300mA for 2h. The PVDF membrane was treated with blotting buffer and incubated at 4°C overnight of different primary antibodies: anti-TGF-β1 (Abcam 1:300), anti-GAP43 (Abcam, USA, 1:500), anti-TH (Abcam, USA, 1:500), anti-NGF (Abcam, USA, 1:400), anti-CaMKII (Abcam, USA,1:2000) anti-PKCδ (Cell signaling, USA,1:1000), anti-Collagen I (Bioss, China,1:200), anti-CollagenIII (Bioss, China,1:200), anti-Smad2 (Bioss, China,1:200), anti-pSmad2 (Bioss, China, 1:200) and anti-β-actin (ZSGB-BIO, China, 1:1500). The PVDF membranes were washed by TBST 3 times and then incubated with horseradish peroxidase-conjugated secondary antibody (Higene, China, 1:2000) for 1h at 37°C. Band intensity was quantified by Quantity One Software (Bio-Rad, Hercules, CA, USA).miR-185 mimics and inhibitor were purchased from RiboBio (Guangzhou, China).

### Patient sample

Total 20 patients diagnosed with COPD or Non-COPD from Hongqi Hospital of Mudangjiang Medical College were involved in this study. 6 pair of normal or COPD lung tissue were collected from autopsy from Mudangjiang Medical College. This study was approved by the ethics committee of Mudangjiang Medical College.

### Cell culture

Human primary pulmonary cell lines, MRC-5 was purchased from American Type CultureCollection (Manassas, VA). Cells were cultured under a humidified air atmosphere containing 5% O2 at 37°C in DME containing 10% FBS.

### Plasmid construction and luciferase reporter assay

Wild-type (WT) or mutant of 3′ untranslated region (Mut) sequences of ColA1 were inserted into the Fse I and Xba I sites of the pGL3 vector (GeneChem). Cells were co-transfected with pRL-TK and collected according to the manufacturer's protocol (Promega)

### Statistical analysis

Animal experiments were repeated at least three times, all data from Sham and OSA group were shown as mean and standard deviation (SD). Student's t-test was used for statistical comparison between groups and p<0.05 was considered as the level of significance.

## Abbreviations

RK: extracellular signal–regulated kinases
CaMKII: the calmodulin-dependent protein kinase-II
PKCδ: protein kinase Cδ
CoLA1: collagen type I
